# Critical Assessment of Novel Developments in HPGR Technology Using DEM

**DOI:** 10.3390/ma17071665

**Published:** 2024-04-04

**Authors:** Victor A. Rodriguez, Gabriel K. P. Barrios, Túlio M. Campos, Luís Marcelo Tavares

**Affiliations:** Department of Metallurgical and Materials Engineering, Universidade Federal do Rio de Janeiro, Rio de Janeiro 21941-972, Brazil; gkpbarrios@metalmat.ufrj.br (G.K.P.B.); tulio.campos@coppe.ufrj.br (T.M.C.); tavares@metalmat.ufrj.br (L.M.T.)

**Keywords:** comminution, high-pressure grinding rolls, simulation, discrete element method, Tavares breakage model

## Abstract

Advances in high-pressure grinding roll (HGPR) technology since its first commercial application in the cement industry include new roll wear protection techniques and new confinement systems. The latter contribute to reductions in the edge effects in an attempt to reach a more homogenous product size along the rolls. Additional advances in this technology have been made in recent years, while modeling and simulation tools are also reaching maturity and can now be used to subject such novel developments to detailed scrutiny. This work applies a hybrid approach combining advanced simulations using the discrete element method, the particle replacement model and multibody dynamics to a phenomenological population balance model to critically assess two recent advances in HPGR technology: spring-loaded cheek plates and the offset roller press. Force and torque controllers, included in the EDEM 2022.1 software, were used to describe the responses of the geometries in contact with the granular material processed. Simulations showed that while the former successfully reduced the lateral bypass of the material by as much as 65% when cheek plates became severely worn, the latter demonstrated lower throughput and higher potential wear but an ability to generate a finer product than the traditional design.

## 1. Introduction

High-pressure grinding roll (HPGR) technology is regarded as one of the most significant innovations in comminution in the last few decades, the success of which is mostly associated with its lower energy consumption, greater amenability of the ore to downstream comminution operations and improved liberation through grain-boundary breakage [1,2,3,4,5]. Application of this inter-particle breakage process in the early 1980s successfully reduced the size of cement clinker, raw meal and diamond ores, all of which are characterized by comparatively low strengths and abrasiveness [3]. However, its first application to hard rock (UCS > 300 MPa) in the Cyprus Sierrita copper concentrator in the United States proved to be a challenge [6], owing to the intensive wear of the rolls and, in consequence, the low mechanical availability of the machine. These challenges were only overcome through innovations in the technology, namely the implementation of rolls fitted with tungsten carbide studs, as well as Hexadur^®^ technology, which dramatically improved the lifetime of rolls in hard rock applications [3,7,8,9].

Another important advancement in HPGR technology is related to the lateral confinement. The main goal of lateral confinement is to reduce the so-called “edge effect”, which consists of dropping the pressure close to the edge of the rolls [10,11], and the clearance distance from the roller edges to avoid the decompression, generating a more uniform product along the length of the rolls. Two types of confinement systems are commercially available. The original is represented by the so-called cheek plates, which remain in use by most HPGR manufacturers. They consist of fixed thrust plates positioned by the sides of the rollers. Metso-Outotec then introduced flanges which consist of rims bolted to the edges of one of the rolls and ensure that the other roll is always under confinement [12,13]. A similar approach is now available from the manufacturer FLSmidth Mining Technologies GmbH (Essen, Germany), being called rotating side plates [14]. More recently, the manufacturer Weir Minerals (Venlo, Netherlands) proposed an improvement in the traditional cheek plate approach. It is called Enduron^®^ and consists of three adjustable spring-loaded elements in the cheek plates: two at the top of the cheek plate system and one at the bottom [15]. According to the manufacturer, the system allows a pre-set minimum gap to be maintained, even after significant wear and roll skewing [15]. 

A more drastic change in HPGR design has been proposed recently by Argo IPS^®^ (Hannover, Germany). It consists of tilting the frame that holds the rollers at an angle of about 30° horizontally while also fitting one of the rolls with flanges. According to the manufacturer [16], this offset placement of the rolls allows them to operate at higher peripheral speeds without slipping. In addition, using a proper control system (AGSL) allows for controlling the effects of the grinding force, throughput and working gap separately. The original design of this HPGR has only one driver, which powers the fixed roll. The floating roll, located in the lower position, rotates as result of its interaction with the particle bed under pressure. Although thought-provoking, no published data are yet available that compare this new design with the traditional HPGR design.

In the last couple of decades, the discrete element method (DEM) has significantly evolved and is now widely accepted as a powerful tool to understand the interaction between the comminution machine and the ore particles [17]. It is a numerical method that has been widely accepted as an effective method to deal with engineering problems involving granular and discontinuous media, with important applications in granular flows, rock mechanics and comminution [17]. This technology, when properly applied and validated, allows for a complete virtual representation of the comminution device [17,18], which is useful in comparing different comminution machine designs and even for conceptualizing an entirely new machine. Indeed, several aspects of the HPGR technology, albeit with simplifications, have already been investigated using DEM. Quist and Evertsson [19] demonstrated that a pressure profile is developed between the rollers. Nagata et al. [20] investigated the effect of different stud configurations on the machine’s performance, while Cleary and Sinnott [21] demonstrated that the level of the feed hopper affects the performance of the HPGRs and also that the solids are not discharged uniformly along the rollers. Additionally, several aspects of the machine’s design and operation have been researched by authors, including the effect of the roll aspect ratio, comparisons of cheek plates and flanges, as well as the performance of HPGRs when operating with rolls exhibiting different wear patterns [22]. In these simulations, the authors described the floating roll motion in one dimension, using the multibody dynamic (MBD) approach implemented as part of an earlier development [23]. An additional advancement in simulation technology was made possible through the description of the motion of the floating roll in two dimensions. This allowed investigations of the phenomenon of skewing that occurs when operating most HPGR designs [24,25,26], demonstrating the differential pressure generated by each piston when attempting to reduce it in the presence of uneven feed [27]. These simulations have been carried out using an effective particle replacement model (PRM), called the Tavares breakage model [28], which describes breakage with a good level of realism. However, given the challenges involved in representing the fine particles in DEM simulations, a hybrid approach which uses a population balance model formulation (PBM) coupled with DEM-MBD-PRM simulations was recently proposed and applied successfully to pilot- and industrial-scale HPGRs [29]. 

The present work follows in the footsteps of earlier work by the authors by using the hybrid approach, that is, a combination of the PBM and DEM, the particle replacement model (PRM) and the multibody dynamics (MBD) model, to study some recent developments in HPGR technology. First, spring-loaded cheek plates are examined through DEM simulations in terms of their capacity, power and working gap. The performance of the HPGR design employing the offset rolls is then analyzed in detail. Then, the alternative designs are compared to simulations of pilot-scale HPGRs with the traditional cheek plates in terms of product size reduction using an iron ore pellet feed.

## 2. Methodology

### 2.1. DEM Simulations

#### 2.1.1. Model Parameters and Base Case Simulation

The material that was the object of the present work is the same as the one used in previous studies by the authors [18,22,27,30] and consists of scaled-up virtual particles representing iron ore pellet feed. The particle size distribution in the feed to the simulated HPGRs had a maximum size of 8 mm (Table 1). Selected material properties are listed in Table 2, whereas contact parameters used in the simulations are listed in Table 3. These have all been estimated on the basis of piston-and-die and bench-scale handling tests [18]. The resultant forces and relative motion of the particles in DEM are calculated using the Hertz–Mindlin (no slip) contact model, and particles are able to break by using a particle replacement strategy [28]. 

The particle replacement model (PRM) was used to realistically represent the response of the bed to the different stress conditions in the DEM simulations. The procedure, based on bench-scale experiments, was also already presented in detail elsewhere [18]. The parameters resulting from that calibration are summarized in Table 4. The parameters $E_{\infty }$, $d_{o}$ and φ define the specific mean fracture energy of the particles, σ is the standard deviation of the log-normal breakage probability distribution, γ represents the amenability of the material to sustain damage and *A* and b′ define the fragment size distribution [28]. *D_min_* represents the minimum size of the particles throughout the simulation. If a particle is predicted to break in the simulation, but one of the fragments is smaller than *D_min_*, the particle becomes unbreakable and its breakage is only accounted for at a post-processing stage [28]. This type of event occurs often in HPGRs, including saturated breakage in the simulation and missing rebreakage, owing to the high stresses reached in the particle bed. As such, the hybrid approach proposed by the authors was used in the present work as an additional strategy to predict the product size distribution [29]. This approach is briefly explained in Section 2.2. 

The DEM simulations were carried out using the software Altair EDEM 2022.1. In this version, force and torque controllers may be linked to the geometries, generating a two-way coupled MBD-DEM model. These controllers have been previously used to calculate the resulting position of the floating roll to simulate traditional HPGRs [18,23]. A force controller in the “x” direction is applied with a kinematic rotational movement. For the fixed roll, only the rotational kinematic movement is necessary since there is no response from the roll because of the interaction with the particle bed. The simulations in the present work only consider one direction of motion of the rolls, and thus did not allow them to skew, unlike in a previous study [27]. 

A pilot-scale HPGR, representing the Roller Press RPR 03.6–100/32 manufactured by KHD Humboldt Wedag AG (Cologne, Germany), with an aspect ratio (diameter/length) of 3.1 (operating parameters are presented in Table 5) and with a filled hopper served as the base case. Changes were then made to this design to represent the novel technologies investigated.

#### 2.1.2. HPGR with Spring-Loaded Cheek Plates (SLCPs)

**Figure 1:**
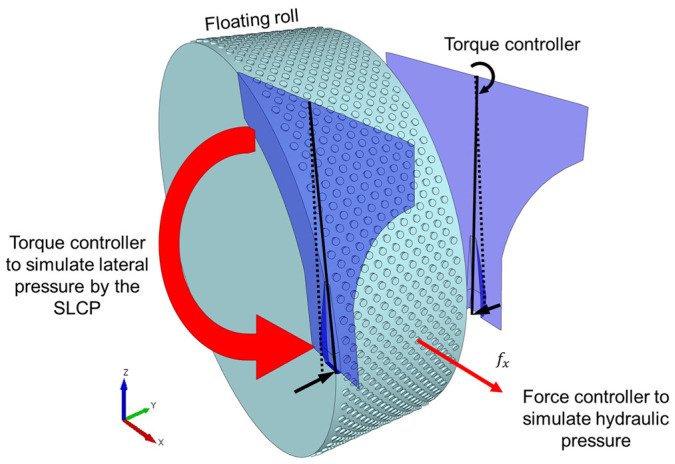
Scheme of the spring-loaded cheek plate (SLCP) in a pilot-scale HPGR simulation using the DEM-MBD-PRM approach. Curved arrows indicate action of torque controller.

The lateral pressure applied by the spring is represented by a torque that allows for opening and closing the bypass gap, that is, the distance between the edge of the rolls and the cheek plate, in response to the interaction with the bed of particles (Figure 1). The descriptions of the floating and the fixed rolls remain the same as those in the base case, already presented in Section 2.1.1 (Table 5). Initially, simulations were carried out to calibrate the torque that needed to be applied to the SLCP to keep the initial bypass gap (i.e., 8 mm) constant when the cheek plates were new. It should be mentioned that, although nowadays check plates may be adjusted down to distances as small as 1 mm from the rolls [15], an 8 mm gap was selected since the particles in the DEM simulations were scaled up [18], as shown in Table 1. 

**Figure 2:**
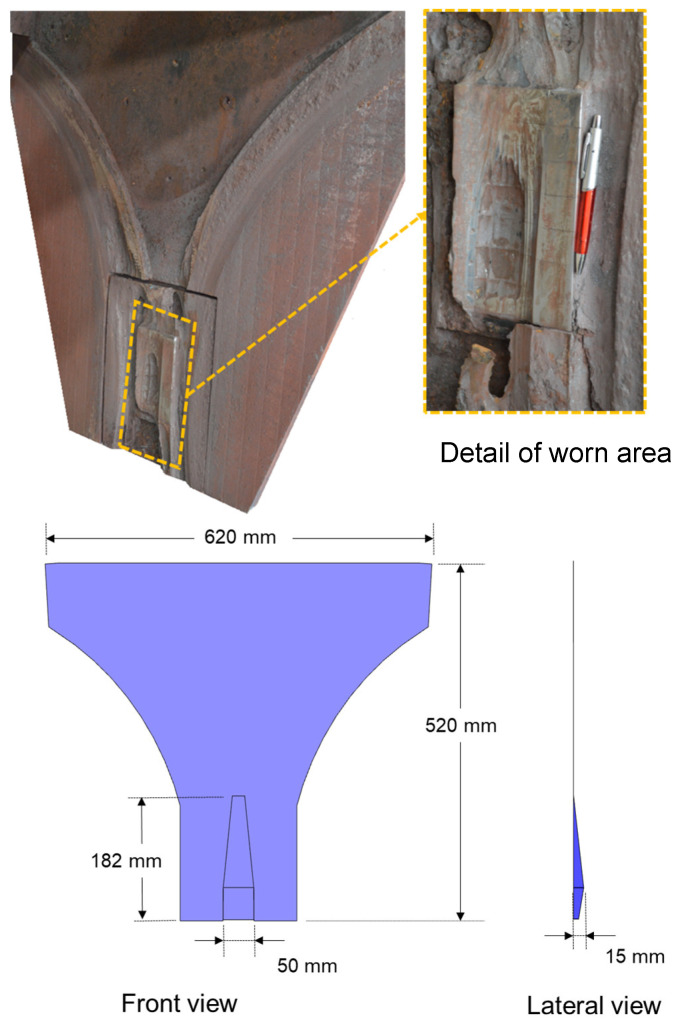
Cheek plates showing the experimental worn pattern (Vale S.A.) (**top**) and CAD design (**bottom**).

Simulations were then carried out for the case when the spring-loaded cheek plates exhibited severe wear in order to demonstrate their ability to compensate for it. In this case, a groove was inserted in the CAD design to represent the wear pattern that was identified in an HPGR operating at one of the operations from Vale S.A. The depth of this groove decreased from the gap to the nip region, reaching a maximum of 15 mm (Figure 2). 

Simulations were carried out with the option of either enabling or disabling the spring loading while the HPGR operated under the same conditions (Table 5), thus allowing the performance of the HPGR with this technology to be assessed. 

#### 2.1.3. Offset Roller Press

**Figure 3:**
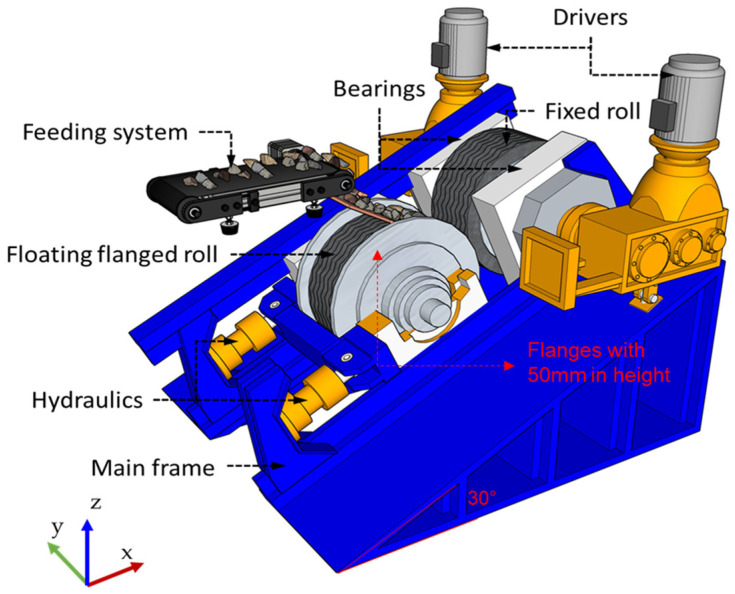
Scheme of the offset roller press. Modified from Drüphake et al. [16].

An HPGR inspired by the design proposed by Argo IPS^®^, as shown in Figure 3, was also simulated. The 30° offset between the rolls was represented by manipulating components in the acceleration due to gravity (Table 6). The operation of this HPGR design used only one driver attached to the fixed roll—as suggested by the manufacturer [16]—and was assessed through a coupled DEM simulation. A zero-value torque controller was included in the center of the mass of the floating roll, increasing the geometry by one degree of freedom and allowing the roll to rotate in response to interactions with the particles. The kinematics of the fixed roll and the force controller used to describe the hydraulic system of the floating roll were kept identical to those of the base case, as described in Section 2.1.3. 

**Figure 4:**
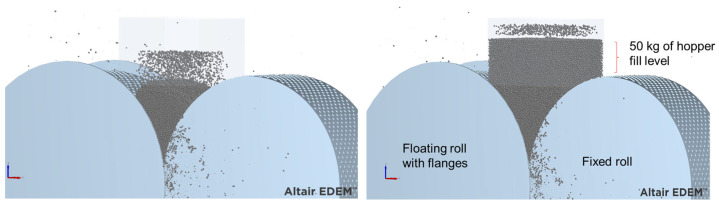
Snapshot of DEM simulations of the traditional HPGR without a hopper (**left**) and with a filled hopper containing 50 kg as in the base case (**right**).

One additional difference in this simulated roller press compared to the base case is the introduction of flanges, measuring 50 mm in height, attached to the floating roller (Figure 3). Also, a simulation of the offset HPGR using an additional driver on the floating roller was also carried out for comparison to the original design in which only one driver is used in the fixed roll. At this point, it is important to recognize that the benefit of the additional pressure associated with the material in the hopper, already evidenced by some authors using DEM to simulate traditional HPGRs [18,21], cannot be taken advantage of by the offset roller press. This is because if a filled hopper is used to feed into the offset rolls, all this pressure will need to be supported exclusively by the floating roll. Thus, to ensure a fairer comparison between the traditional HPGR and the offset roller press, simulations of the HPGR of the traditional design were conducted not only with a hopper filled (choke-feeding) with 50 kg of ore, which approximates its usual operation, but also with the minimum level of ore needed to fill the particle bed between the rolls in order to mimic the offset HPGR design. Figure 4 presents snapshots of the DEM simulations with a traditional HPGR without a hopper and with a filled hopper as described in the base case.

#### 2.1.4. Measurement of Key Variables

To compare the simulation results and evaluate the improvements from the innovative designs, some key pieces of information were extracted. Since the approach used is identical to the one already used in earlier publications [18,22], it is only described briefly, as follows. The power of the HPGR during operation was calculated from the torque measured using the DEM simulation multiplied by the roll velocity. Also, a mass flow sensor was used to measure the total throughput of the HPGR product. To analyze the variation in mass flow as a function of roll length, sixty bins were positioned underneath and along the length of the rolls in the discharge zone. To analyze the force profile, bins were positioned in the compression zone, that is, between the working gap and the nip angle. Such profiles are presented as a function of the rolls’ length considering the y-axis identified in Figure 1 and Figure 3.

### 2.2. Hybrid Model

In the present work, prediction of the product size distribution was carried out using the hybrid model recently proposed by the authors [29]. The model consists of linking the outputs of the DEM-PRM-MBD simulations with the population balance model (PBM) [29]. DEM outputs such as total throughput and power, as well as information on the pressure and throughput profile along the rolls’ length are used in the PBM to predict the product fineness. The approach was able to predict the product particle size distributions of a pilot-scale HPGR operating at different roll velocities and pressures in size reduction [23]. In the PBM, two functions need to be fitted from the experimental data: the breakage (*B*) and the specific selection function ($s_{i}^{E}$) [29,31]. The parameter *B* defines how the fragments provided by a broken particle are distributed in different size classes, while the parameter $s_{i}^{E}$ allows calculating the breakage rates. This fitting was performed on the same pilot HPGR unit operating under the same conditions as the base case described in Section 2.1.1. The resulting parameters, listed in Table 7, have been validated on both pilot- and industrial-scale HPGRs [29]. Both functions were assumed to remain the same for the different HPGR designs, with the breakage rates varying only as a function of their power and throughput. 

## 3. Results and Discussion

### 3.1. Spring-Loaded Cheek Plates (SLCPs)

**Figure 5:**
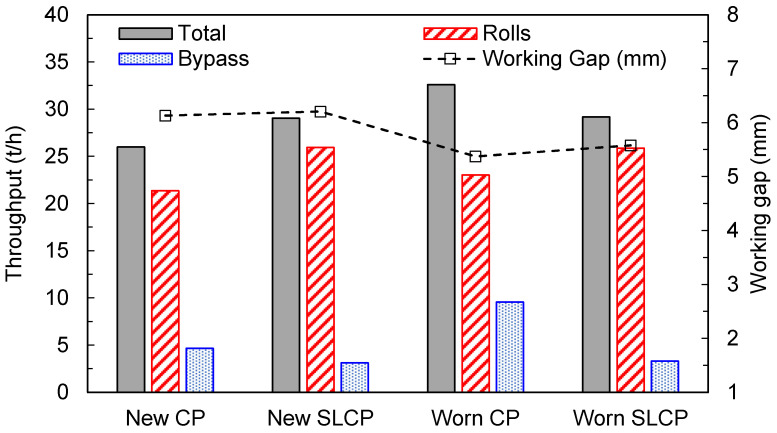
Predictions of throughput (total, between the rolls and bypass) and working gap of the pilot-scale HPGR simulated with new and worn cheek plates (CPs) and spring-loaded cheek plates (SLCPs). The machine operates at 3.5 N/mm^2^ with a rotational roll velocity of 0.5 m/s.

A first comparison of the performance of the SLCPs to that of the original design is shown in Figure 5 for the total throughput. The figure also differentiates the material passing through the rolls (identified as “Rolls” in the figure) from that bypassing the rolls and being discharged from the edges (named “Bypass” in the figure). In this condition, a torque of about 50 kN∙m from a point of action of 0.45 m was applied to the SLCPs to constantly maintain the bypass gap at about 8 mm. The use of SLCPs shows a marginally higher throughput when the CPs are new, mostly associated with the material passing through the rolls (~12%), since the flowrate of the bypassing material is reduced by nearly 65%. This might be explained, at least in part, by the lateral pressure exerted by the SLCPs when they are new, which increases the pressure on the particle bed in direct contact with the rolls. This pressure is also responsible for the minor increment observed in the operating gap of the HPGR when using SLCPs in comparison to CPs when both are unworn (Figure 5). This trend was also observed in the experimental pilot-scale tests carried out with and without the SLCPs [15]. In those experiments, the authors observed a 2.5% relative increase in the working gap and increases between 3 and 20% in the flowrate of materials passing through the rolls in three of the four tests as a result of the use of SLCPs. 

When the traditional fixed cheek plates exhibit severe wear (“Worn CP” in Figure 5), a significant increase in throughput occurs, mostly associated with the increase in the flowrate of the material ejected from the edges of the rolls (bypass), which more than doubled, with an increase from 4.7 to 9.6 t/h. A reduction in the working gap, which was the response of the system to maintaining a constant specific force, was also observed. On the other hand, operation of the roll with severely worn SLCPs did not significantly affect either the total throughput or the flowrate of material being ejected at the edge of the rolls. As observed when comparing simulations of new and worn CPs with simulations of new and worn SLCPs, a reduction in the working gap was also identified.

**Figure 6:**
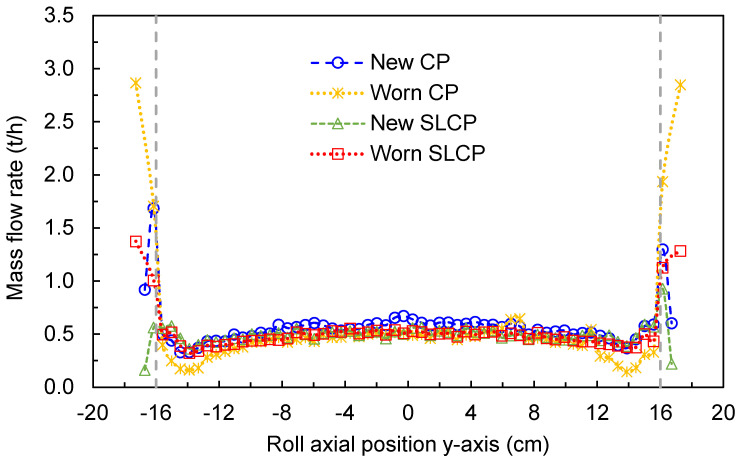
Average axial mass flowrate along the rolls in a pilot HPGR with different confinement conditions operating at 3.5 N/mm^2^ and rotational roll velocity of 0.5 m/s. Roll axial position is defined as in Figure 1. Vertical dashed lines represent the length of the rolls.

Additional insights can be gained by analyzing the flow of the material along the length of the rollers, as shown in Figure 6. The results shown in Figure 6 are consistent with those summarized in Figure 5, but highlight the increase in the amount of material bypassing the rolls when the cheek plates are worn. This also results from the actual reduction in the flowrate in the region immediately before the edges, a reduction that is controlled by using the SLCPs (red line Figure 6). Finally, the use of SLCPs when the cheek plates are new showed very low bypass flowrates, which slightly increased after the SLCPs became worn.

**Figure 7:**
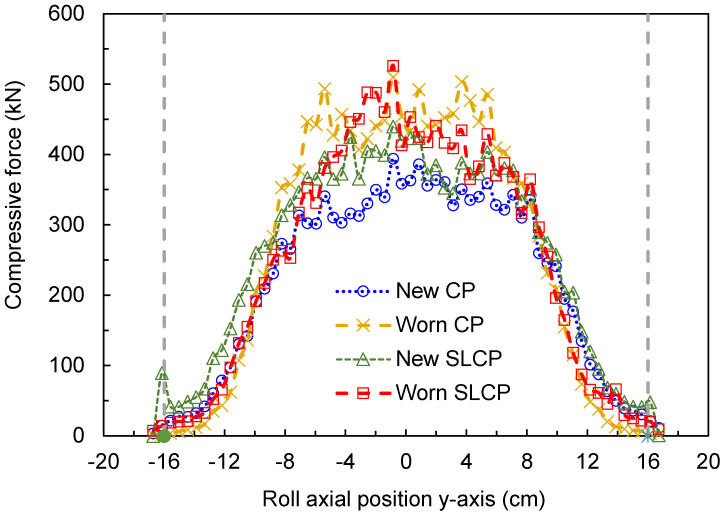
Predicted force profile along the rolls of a pilot HPGR with different confinement conditions, operating at 3.5 N/mm^2^ and a rotational roll velocity of 0.5 m/s. Roll axial position is defined as in Figure 1. Vertical dashed lines represent the length of the rolls.

One of the most important pieces of information that can be obtained from a DEM simulation of an HPGR is how the pressure/force is distributed along the rolls. This information allows the edge effect to be assessed, which is the main target of the confinement system. Figure 7 compares the compressive force profiles for the HPGRs with the standard CPs and the SLCPs, both new and worn. It shows that the operation of an HPGR that exhibits wear in the confinement system results in higher forces in the center of the rolls, with a significant edge effect, which would likely translate to a more heterogeneous product size along the rolls. Marginally higher pressures along the rolls are observed when comparing the SLCPs and CPs when both are new, but similar force distributions are observed once the confinement systems become worn, with the SLCPs exhibiting slightly higher pressures at the edge. Although the use of the SLCPs showed a reduction in bypassed material, the parabolic pressure profile remains, as observed when new traditional CPs were used [18,30]. Nevertheless, it is worth mentioning that the HPGR operating with worn CPs demonstrated a more trapezoidal shape profile.

**Figure 8:**
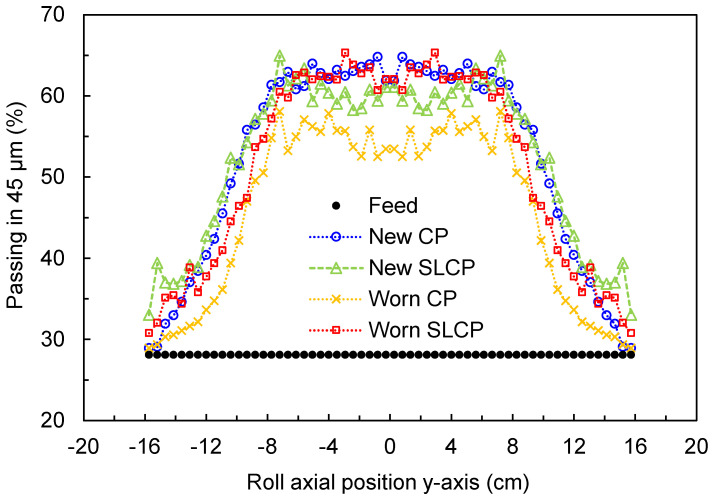
Prediction of variation in percentage of material passing the 45 μm sieve along the axial roll position using the hybrid model for simulations of the pilot-scale HPGR performed under different lateral confinement conditions. Simulations used a specific compressive force of 3.5 N/mm^2^ and a rotational roll velocity of 0.5 m/s. Roll axial position is defined as in Figure 1.

The hybrid model was then used with the aim of comparing the axial variation in the product size distribution along the length of the rolls [29]. This approach combines information from the axial variation in the force and mass flowrate, obtained in the coupled DEM simulation, and the energy-specific selection function from the population balance model [31], previously calibrated on the basis of data from a base case. The results are shown in Figure 8, where the percentages of material passing the 45 µm sieve along the axial roll position are compared across simulations performed using different lateral confinement conditions. A significant reduction in the percentage of material passing the 45 µm sieve along the roll length is evident when comparing the pilot-scale HPGR operating with new and worn cheek plates. Besides the difference in magnitude, both profiles can be discriminated by their shape, in which the HPGR with new cheek plates presented a nearly parabolic shape with a smooth edge, as already observed elsewhere [18,19,30], whereas the HPGR with worn cheek plates clearly presented a trapezoidal profile with a severe drop in size reduction towards the edge of the rolls. The net result was a reduction in the percentage of material passing through the 45 µm sieve, which dropped from 51.9% when the CPs were new to 50.2% after they were worn.

As an alternative to the new cheek plates, Figure 8 also presents the percentage of material passing the 45 µm sieve along the axial roll position when simulating the HPGR equipped with spring-loaded cheek plates, which demonstrated a reasonable increment in size reduction close to the edge of the rolls. As demonstrated elsewhere through experimental tests [15], the use of spring-loaded cheek plates can prevent feed material from being ejected at the edge of the rolls, which is often observed during HPGR operation [18,30]. This would, in turn, translate into an ability to maintain the quality of the final product. Comparisons between new cheek plates and new spring-loaded cheek plates also demonstrated a similar profile along the axial roll position. However, in simulations, worn SLCPs were more capable than worn CPs when approaching the same breakage intensity in the center region of the roll obtained when the CPs were new, without the significant drop that occurs towards the edges when the CPs become worn. 

A summary of the results is presented in Table 8. At first, a comparison of the systems when dealing with the new cheek plates shows that the torque/pressure exerted by the SLCPs on the particle bed to keep the bypass gap relatively unchanged resulted in an increase in the torque required to rotate the rolls. This becomes evident from the higher power and specific energy demanded by the machine. These results are also in agreement with results from Van der Ende [15], who reported that the action of the SLCPs increased the power and specific energy of the HPGR by around 7% and 34%, respectively. Now, comparing the power demand of the HPGR using different cheek plates as they wear, in the case of the original design, the power is increased due to the reduction in the gap, but the throughput is increased even further, resulting in a reduction in specific energy (Wc). In the table, it is also evident that the use of the SLCPs allowed the product fineness to be kept nearly constant, in contrast to the CPs, which exhibited an important reduction in fineness as the cheek plates became severely worn. 

### 3.2. Offset Roller Press

**Figure 9:**
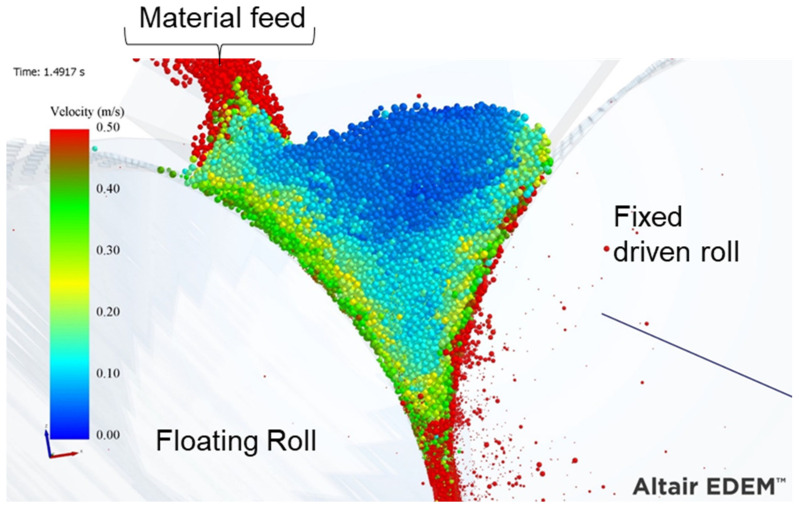
Image of simulation of the offset roller press with particles colored according to their velocity. Roller press operating at a specific force of 3.5 N/mm^2^ and peripheral roll velocity of 0.5 m/s.

A snapshot of the offset HPGR simulation with a single driver is presented in Figure 9. Particles are colored according to their velocity, revealing that particles at the top of the particle bed are nearly stationary and experience acceleration as they are transported to the maximum pressure zone. The particles in contact with the fixed roll are transported at the same velocity as the roller (0.5 m/s) due to the action of the studs, which prevent slipping on the roller surface. Cleary and Sinnot [21] observed a reduction in velocity for these particles; however, this was likely because those authors used smooth rollers. In contrast to that work, a reduction in particle velocities with lower values in the central layers is evident in Figure 9. Due to the confinement conditions, the velocities of particles that are close to the floating roll are higher than those in the central layer, but all are smaller than 0.5 m/s, which is the peripheral velocity of the roll and the particles directly attached to it. This interaction increases the friction and shear between layers, a response that could result in a potential benefit regarding breakage.

**Figure 10:**
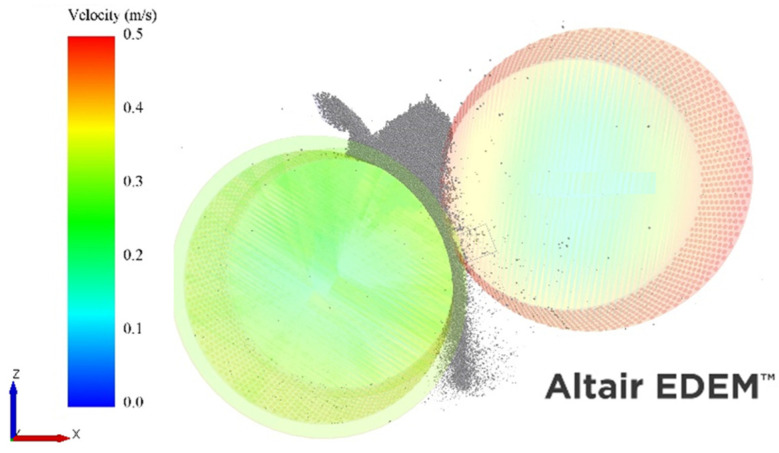
Image of the DEM simulation of the offset roller press with a single driver with geometries colored according to their velocities. Roller press operating at a specific force of 3.5 N/mm^2^ and a fixed driven roll velocity of 0.5 m/s.

Figure 10 shows the velocities of the rollers in the simulations. It is evident that the transfer of momentum to the floating roll is inefficient, as the peripheral velocity of the floating roller is nearly 40% lower than that of the driven roller. 

**Figure 11:**
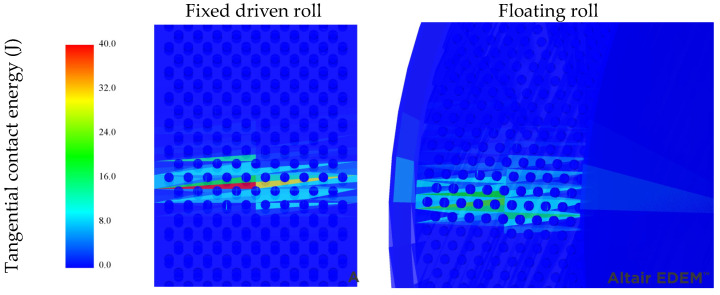
Tangential contact energy (J) in the compression zone in a time step of the simulation of the floating and fixed driven rolls of the offset HPGR operating at 3.5 N/mm^2^ and a peripheral speed of 0.5 m/s for the fixed roll, predicted using DEM.

One potential outcome of this would be dissimilar wear on each roll. This is examined in Figure 11, where the mesh of each roll is colored according to its tangential contact energy in a time step once the machine was in steady state. The results reveal that higher tangential contact energy is dissipated on the fixed driven roll. From this finding, it may be inferred that there will likely be greater wear on the fixed roller than on the floating roller. In the traditional HPGR, higher wear in the floating roller has been reported [32,33,34,35], generating differences in the rollers’ peripheral velocities due to the uneven reduction in diameter. This condition accelerates wear once the tangential forces between the roller and ore increase. However, in this case, where only the fixed roll is driven, the increase in wear rates owing to the reduction in diameter will likely not occur, unless studs were removed from the roll.

**Figure 12:**
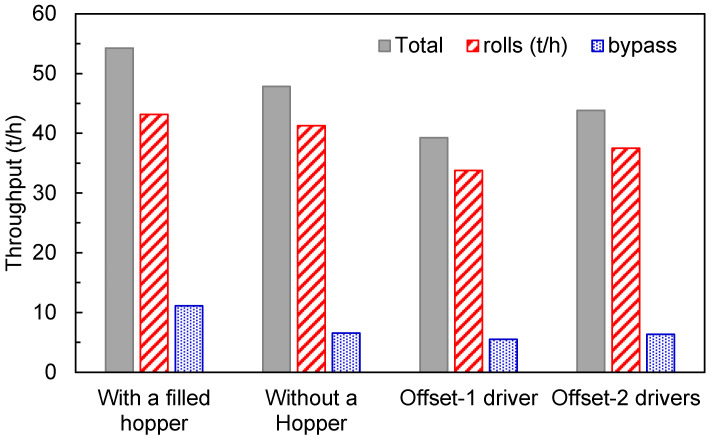
Predictions of throughput of the traditional HPGR (with a filled hopper) compared to results of simulations of the traditional HPGR without a hopper and the offset roller press with single and twin drivers operating at 3.5 N/mm^2^ and 0.5 m/s.

**Figure 13:**
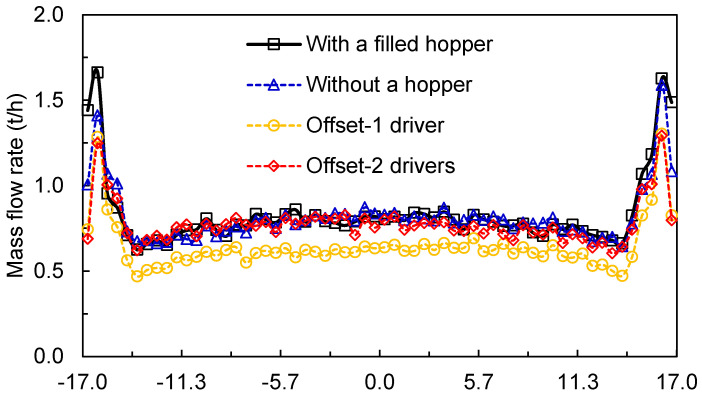
Predicted average axial mass flowrate along the rolls in a pilot HPGR with different configurations operating at 3.5 N/mm^2^ and rotational roll velocity of 0.5 m/s. Roll axial position is defined as in Figure 1 and Figure 3.

The results of the key variables for each condition are discussed as follows. Figure 12 presents the throughput, discriminated by the material transferred by the rolls and bypass. The performance of the offset roller when operating with single and twin drives is compared to an HPGR with the traditional design (base case) with 50 kg of material in a hopper chocking it (filled hopper). The DEM simulation predicted higher throughput for the traditional choke-fed HPGR, whereas a smaller mass transfer is predicted when using one driver in the offset roller press. Material is ejected at similar flowrate at the bypass in almost all of the conditions compared, except for the filled hopper condition, which showed an increase of 43%. The mass flow rate along the rolls is presented in Figure 13, revealing consistent results, with a smaller value for the offset HPGR with one driver. 

**Figure 14:**
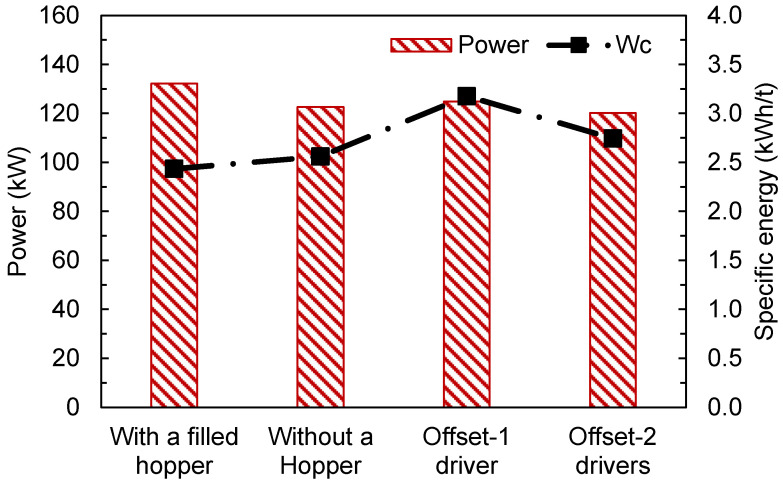
Predicted overall power and specific energy consumption (*W_c_*) of traditional and offset pilot-scale HPGRs with different configurations operating at 3.5 N/mm^2^ and a rotational roll velocity of 0.5 m/s.

The values of the overall power and specific energy consumption (Wc) are summarized in Figure 14. The highest power was predicted in the case of traditional HPGR (with the filled hopper), but since it has also the highest throughput, it had the lowest specific energy consumption. Using only one driver in the offset roller resulted in the highest specific energy consumption among all conditions simulated, as well as a higher power than the traditional HPGR without a hopper and the offset roller using two drivers. This may be explained by the fact that a portion of that energy is used to slip the layers between the rollers (Figure 9), which may present an advantage in comminution owing to the increase in particle–particle friction while the material struggles to pass between the rolls.

**Figure 15:**
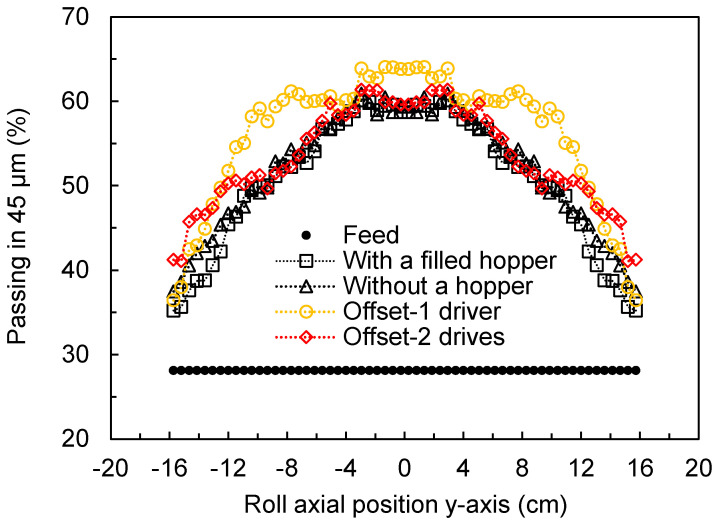
Predicted variation in percentage of material passing through the 45 μm sieve along the axial roll position predicted using the hybrid simulation model performed using different configurations of traditional and offset HPGRs. Simulations used a specific compressive force of 3.5 N/mm^2^ and rotational roll velocity of 0.5 m/s. Roll axial position is as in Figure 1 and Figure 3.

An additional analysis using the hybrid model is presented in Figure 15, which compares the percentage of material passing through the 45 µm sieve along the axial roll position under the conditions studied. A finer product and a nearly trapezoidal profile with a more aggressive drop can be seen when the offset HPGR operates with a single driver. This may be explained by its higher power values (Figure 14) and lower throughput (Figure 12), since the breakage rates in the hybrid model are directly related to their ratio [29,31]. For the rest of the scenarios, Figure 15 shows parabolic profiles with a smooth drop in breakage intensity towards the edge of the rolls.

Finally, Table 9 summarizes the main results of the coupled DEM simulations and hybrid model predictions. The operation of the HPGR with a filled hopper was shown to be beneficial as it processed more material with less power. However, this mode of operation resulted in higher bypass rates towards the edges, due to the higher pressure drop; this also resulted in the coarsest product among the scenarios simulated. On the other hand, the operation of the offset HPGR resulted in a lower throughput than the traditional design, even when compared to the HPGR with no hopper. In addition, the offset HPGR operated with smaller gaps but with comparable powers. The result was an increase in the fineness of the product, as evidenced by the percentage of material passing through the 45 μm sieve. Table 9 also shows that no clear benefits of using an additional driver exist from the standpoint of the performance variables studied, confirming the design proposed by the manufacturer [16].

### 3.3. Analysis from a Materials Perspective

Surface wear in HPGRs is well-known for causing losses in operating time and machine performance. Typically, surface wear patterns within HPRGs follow the pressure profile along the roll length, thus providing a more pronounced wear in the center of the rolls, often with parabolic and trapezoidal shapes [36]. The prevention of roll surface wear involves proper material selection [9], surface wear protection designs [37] and understanding the different wear patterns formed along the rolls, which have been achieved in the present work and in previous works by the authors [22]. In this regard, the results presented in Section 3.1 and Section 3.2 show the different regions along the roll length that are more susceptible to significant roll surface wear when analyzing the different technologies. Operating an HPGR with non-uniform force profiles, as depicted in Figure 7, would result in uneven wear on the material surface along the rolls’ lengths. These results assist, at least partially, in the selection of proper materials and the proposal of suitable designs of the roll surface and lateral confinement systems to prevent the occurrence of the heterogenous wear profiles. In addition, as discussed in Section 3.2, Figure 11 shows a higher energy dissipation on the fixed roll of the offset HPGR, which suggests a higher wear and potentially higher loss of material from the roll’s surface. 

As such, the present work showed that DEM simulation of HPGRs may be a useful tool to assess new technological developments proposed by manufacturers, mainly aiming to improve the performance of the wear of materials on the roll’s surface but aligned with the need to maintain or improve the processing performance of the machine.

## 4. Conclusions

Novel developments in HPGR technology were studied through simulations using the coupled DEM-MBD-PRM method in association with a hybrid model based on the population balance model to predict key variables as well as product size distribution.

A pilot-scale HPGR equipped with spring-loaded cheek plates (SLCPs) was simulated both when the cheek plates were new and after undergoing severe wear; equivalent simulations were conducted using fixed (traditional) check plates (CPs). The results demonstrated the benefit of this technology since a reduction in the edge effect and increase in confinement were evident. Higher power and specific energy were observed as a result of using the SLCPs, with a finer product predicted using the hybrid model. Simulations indicated that severe wear of the traditional cheek plates would be detrimental to the HPGR’s performance, but the application of SLCPs would allow both the fineness of the product and the machine throughput to be kept nearly constant.

The offset HPGR was also simulated, and its performance was compared to the HPGR following the traditional design. Simulations using a driver only in the fixed roll, as proposed by the manufacturers, and with two drivers were analyzed and compared with simulations of the traditional HPGR with a filled hopper and without a hopper to allow a proper comparison. The results demonstrated the validity of using a single driver. Although a higher power and a lower specific energy consumption were observed, the use of a driver for only a single roll generates slippage within the bed of particles, which is a potential benefit to comminution. The effect of using a filled hopper in the traditional HPGR increased the material’s mass flow and resulted in a higher power draw by the machine. The operation of the traditional HPGR with a filled hopper indeed proved to be more beneficial when compared to the remaining simulated scenarios when considering throughput and power. In contrast to that, the offset HPGR with a single driver resulted in the finest product. However, this configuration may have the detrimental effect of increasing the wear of the rolls, especially in the case of the fixed driven roll. 

This work demonstrates how simulation technology, with a few assumptions and with proper calibration, may be successfully used to analyze novel HPGR designs. 

## Figures and Tables

**Table 1:** Particle size distribution used as the feed in the simulations [18].

Particle Size (mm)	8.0	7.2	6.4	5.6
% in volume	40	23	22	15

**Table 2:** Selected material properties used in the simulations [18].

Density of DEM Particles (kg/m^3^)	Experimental Bulk Density (kg/m^3^)	Shear Modulus (GPa)
3011	3010	2.0

**Table 3:** Hertz–Mindlin contact parameters used in the simulations [18].

Coefficient	Ore–Steel	Ore–Ore
Restitution	0.15	0.20
Static friction	0.49	0.55
Rolling friction	0.47	0.51

**Table 4:** Breakage parameters used in the simulation [18].

Parameter	$E_{\infty }$ (J/kg)	$d_{o}$ (mm)	φ	σ	γ	A (%)	b′	*D_min_* (mm)	*E_max_*/*E*_50_
Value	500	75	0.35	0.8	5.0	68	0.03	1.2	4

**Table 5:** Summary of the main design and operating conditions of the pilot-scale HPGR (base case).

Variable	Unit	Value
Roll diameter	m	1.0
Roll length	m	0.32
Specific force	N/mm²	3.5
Rolls velocity	m/s	0.5
Initial gap	mm	5
Bypass gap	mm	8
Confinement system	-	Cheek plate

**Table 6:** Component of the gravitational force used in the offset roller press simulations.

Component	Gravity (m/s^2^)
x	−4.90
y	0
z	−8.49

**Table 7:** Fitted selection and breakage function parameters for the hybrid model [29,30].

Equation	Parameter	Value
Breakage function (*B*)	γ	0.896
	β	5.461
	ϕ	0.857
	η	0.46
	ω (mm)	0.013
Specific selection function ($s_{i}^{E}$)	$\xi_{1}$	−0.492
	$\xi_{2}$	−0.190
	$s_{1}^{E}$ (t/kWh)	0.247

**Table 8:** Summary of the results of DEM simulations for different lateral confinement conditions in the HPGR.

Configuration	Throughput (t/h)			Power (kW)	Working Gap (mm)	Wc (kWh/t)	Material Passing 45 μm (%)
	Rolls	Bypass	Total				
New CPs	21.3	4.7	26.0	70.7	6.13	2.72	51.9
New SLCPs	25.9	3.1	29.0	88.2	6.21	3.04	55.1
Worn CPs	23.0	9.6	32.6	77.7	5.38	2.38	50.2
Worn SLCPs	25.9	3.3	29.2	88.2	5.58	3.02	54.6

**Table 9:** Summary of results of DEM simulations of the traditional and offset HPGRs.

Configuration	Throughput (t/h)			Power (kW)	Working Gap (mm)	W_c_ (kWh/t)	Material Passing 45 μm (%)
	Rolls	Bypass	Total				
HPGR—filled hopper	43.2	11.1	54.3	132.2	13.9	2.4	50.7
HPGR—no hopper	41.3	6.6	47.9	122.6	13.9	2.6	51.8
Offset—1 driver	37.5	6.3	43.8	120.2	7.2	2.7	56.2
Offset—2 drivers	33.8	5.5	39.3	124.9	8.5	3.2	53.3

## Data Availability

Data are available upon request.
